# Tuneable Q-Factor of MEMS Cantilevers with Integrated Piezoelectric Thin Films

**DOI:** 10.3390/s18113842

**Published:** 2018-11-09

**Authors:** Martin Fischeneder, Martin Oposich, Michael Schneider, Ulrich Schmid

**Affiliations:** Institute of Sensor and Actuator Systems, TU Wien, 1040 Vienna, Austria; martin.oposich@aon.at (M.O.); michael.schneider@tuwien.ac.at (M.S.); ulrich.e366.schmid@tuwien.ac.at (U.S.)

**Keywords:** electronically tuneable Q-factor, MEMS cantilever, peak shaping, AlN, piezoelectric, phase shifted excitation, AFM, vacuum

## Abstract

In atomic force microscopes (AFM) a resonantly excited, micro-machined cantilever with a tip is used for sensing surface-related properties. When targeting the integration of AFMs into vacuum environments (e.g., for enhancing the performance of scanning electron microscopes), a tuneable Q-factor of the resonating AFM cantilever is a key feature to enable high speed measurements with high local resolution. To achieve this goal, in this study an additional mechanical stimulus is applied to the cantilever with respect to the stimulus provided by the macroscopic piezoelectric actuator. This additional stimulus is generated by an aluminum nitride piezoelectric thin film actuator integrated on the cantilever, which is driven by a phase shifted excitation. The Q-factor is determined electrically by the piezoelectric layer in a Wheatstone bridge configuration and optically verified in parallel with a laser Doppler vibrometer. Depending on the measurement technique, the Q-factor is reduced by a factor of about 1.9 (electrically) and 1.6 (optically), thus enabling the damping of MEMS structures with a straight-forward and cheap electronic approach.

## 1. Introduction

In recent years a large number of silicon based MEMS (micro electro-mechanical systems) sensors and actuators were developed. Besides a high technology readiness level, this success is based on the broad range of different application scenarios covering sensors for e.g., the detection of chemical [1,2] or physical quantities [3,4], what requests, however, an individual and an application-specific design. But, despite any differences, most approaches make use of either membranes or cantilevers as functional key components. Furthermore, many MEMS devices are operated in resonance by applying either electro-magnetic, electro-thermal [5,6], capacitive or piezoelectric actuators for excitation [7] to increase the sensitivity.

When making use of the latter transducer principle, a typical design consists of a piezoelectric aluminum nitride layer (AlN) sputter deposited on a released silicon (Si) support structure clamped to a substrate [8]. Although offering only moderate piezoelectric constants [9], AlN is often preferred compared to zinc oxide (ZnO) or lead zirconate titanate (PZT), as it is compatible with standard complementary metal oxide semiconductor (CMOS) microfabrication processes [10] and offers a high temperature stability [11]. Cantilever or membrane-type micro-machined AlN devices are most promising candidates for density and viscosity sensors of liquids [12,13], high frequency filters [14], MEMS scanning mirrors [15] or vibrational energy harvesters [16,17].

Advanced future analyses of complex surfaces require an extensive characterization by measuring a large variety of topography and material-related parameters, which are provided by the sophisticated combination of well-established techniques such as scanning electron microscopy (SEM) and atomic force microscopes (AFM). Doing so provides the possibility to investigate the same area of interest with both SEM and AFM. However, standard AFM cantilevers excited by a piezo-shaker feature a low bandwidth in vacuum due to increased Q-factors as the damping by the surrounding atmosphere is negligible compared to a standard operation in air [18]. To overcome this drawback, the realization of an electronically adjustable Q-factor is targeted to improve the performance of resonantly operated AFM cantilevers.

A common method for active modification of the Q-factor is the implementation of a feedback on the tapping piezo based on the optical beam deflection [19]. The improvement of the image quality by mixing a 90° phase shifted signal to the cantilever deflection signal is shown in [20]. By removing the optical sensor from the feedback loop, an active shunt replaces the deflection measurement with a tip velocity measurement. The piezoelectric layer is driven by a synthetic impedance [21] and reduces the Q-factor. The use of the electrical cantilever velocity signal for a feedback loop necessitates the compensation of parasitic effects, as shown in [22]. A tailored differential sensing approach is used to cancel out manufacturing tolerances. By adding various active layers for actuation and sensing purposes, a positive position feedback (PPF) controller and a field-programmable analogue array (FPAA) are implemented as a multimode Q-controller that adjusts the Q-factor within three to four orders of magnitude [23].

It is the objective of this study to determine the potential of active Q-factor tuning with one piezoelectric element integrated on a MEMS cantilever [24]. For demonstration purposes, a tailored electrical circuit stimulates the thin film actuator with a frequency-dependent phase shift, thus manipulating the oscillation of the cantilever which in turn is excited by the macroscopic piezoelectric actuator as it is usually done in AFMs. In contrast to other technically demanding techniques, the implementation of a straightforward approach is in the focus of this work by only using standard lab-equipment for the manipulation of the cantilever oscillation. Therefore, the straight-forward transfer to a microprocessor controlled unit is feasible, so that a low-cost add-on electronic system can be realized, which can be placed close to the cantilever for minimizing signal losses.

## 2. Experimental Details

**Figure 1:**
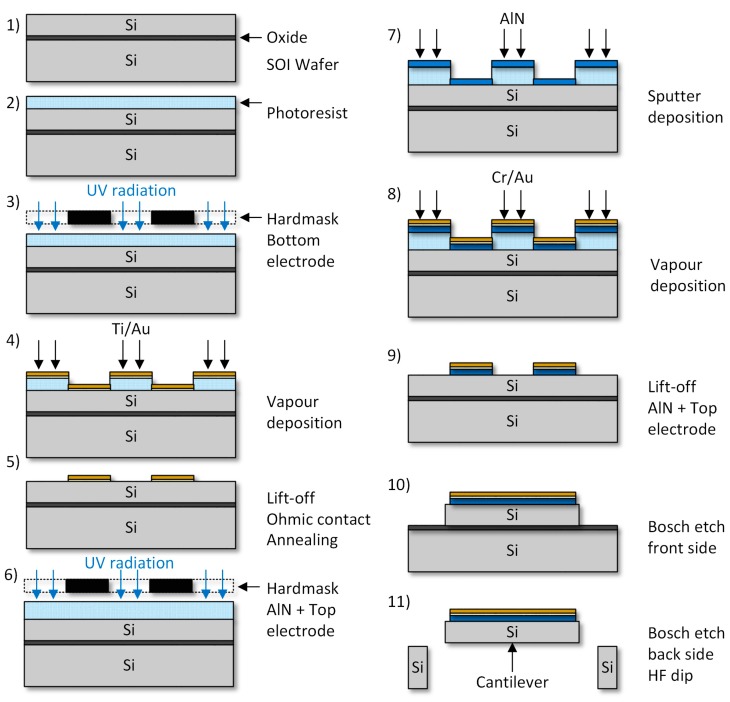
Schematics of the fabrication process.

For hardware realization, a fabrication process was developed based on 4-inch silicon-on-insulator (SOI) (0.5 µm buried oxide) wafers, as shown in Figure 1. The device layer with a thickness *T_Sub_* of 20 µm is highly boron p-doped (0.01–0.02 Ω·cm) serving as bottom electrode on the cantilever. The surface is covered with a reactively sputter deposited aluminum nitride (AlN) thin film (AlN thickness Δ = 500 nm), as shown in Figure 1, steps 1–4. The AlN thin film is deposited with an industry type DC magnetron sputter equipment (Von Ardenne LS730S, Dresden, Germany). During AlN synthetization the wafer is continuously self-heated by the particle bombardment. In order to ensure a low sample temperature (T < 140 °C), thus avoiding the degeneration of the photo-resist and enabling a lift-off process for AlN patterning (Figure 1, step 5), a tailored clamping fixture for the 4” wafers was used [25]. After deposition and patterning of the gold (Au) top electrode (top electrode thickness *T_TE_* = 200 nm) the piezoelectric thin film actuator stack is completed, as shown at Figure 1, steps 6–9. By applying a Bosch etch process at front and back side the cantilever is defined and released (Figure 1, steps 10 and 11).

**Figure 2:**
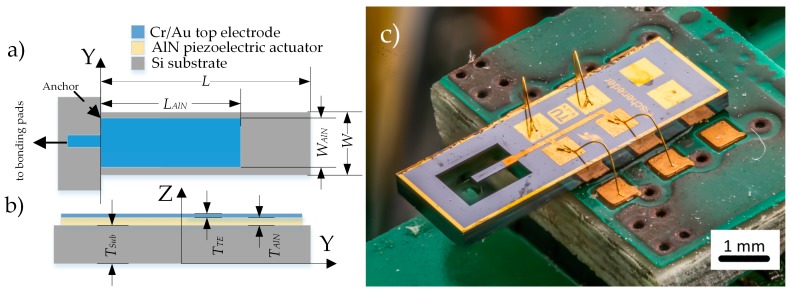
Schematics of the piezoelectric micro electro-mechanical systems (MEMS) cantilever. In (**a**) the top, in (**b**) the cross-sectional view and in (**c**) micrograph of the cantilever are shown.

The cantilever in Figure 2 was designed having a resonance frequency of the first bending mode lower than the critical frequency of the measurement equipment (f < 100 kHz), which is limited by the shaker piezo design. Basically, the cantilever has a length of *W* = 750 µm, a width *W* = 160 µm and is covered with AlN by a length *L_AlN_* = 200 µm, a width *W_AlN_* = 154 µm resulting in a resonance frequency of 48.5 kHz in air.

**Figure 3:**
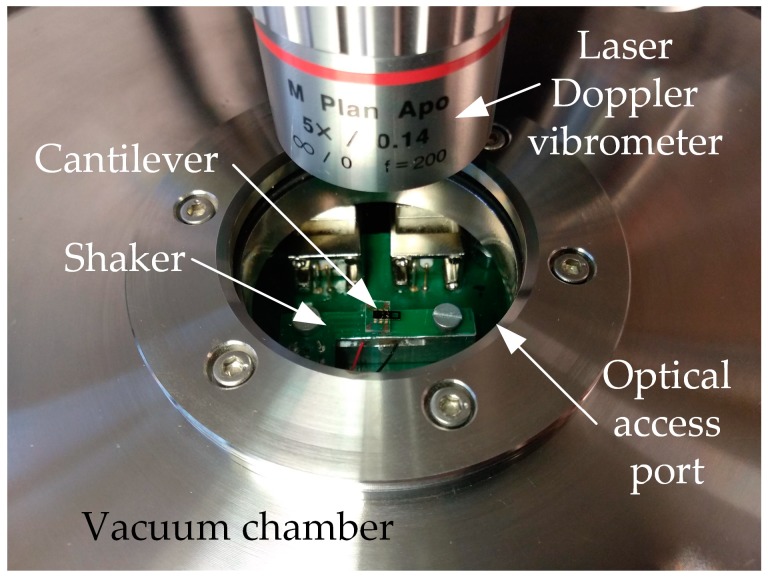
Photograph of the bonded MEMS cantilever mounted on a custom-built shaker. The complete set-up is placed in a vacuum chamber having optical access for the laser Doppler vibrometer (LDV) measurements.

The fabricated cantilever is glued and bonded to a specially designed printed circuit board (PCB) which connects the cantilever electrically to the electrical stimulus and mechanically to the piezo shaker. The optical photograph in Figure 3 shows the measurement setup. To demonstrate the loss of the damping, the cantilever and the PCB are exposed to a pressure of 6.5 × 10^−5^ mbar in a specific vacuum chamber, so that viscous damping effects of the residual gas atmosphere are negligible [26].

**Figure 4:**
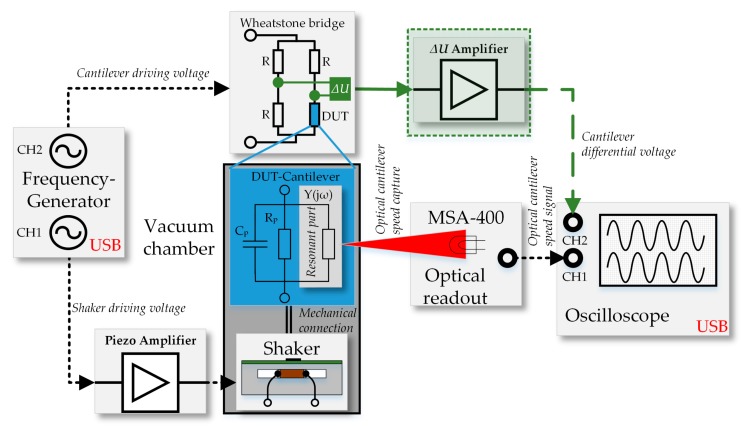
Block diagram of the measurement set-up used for characterization of the actively damped MEMS cantilevers. The electrical readout is highlighted in green.

The vacuum chamber has both, an optical access port and electrical feedthroughs which connect the PCB with the cantilever to the driving and measurement equipment. The complete measurement system is illustrated in Figure 4 and is controlled by a MATLAB script via an USB interface.

A discrete set of sinusoidal frequencies (frequency spectrum) without DC offset is provided at CH1 and CH2 of the frequency generator (FG). The signal of FG–CH1 is boosted by a custom-made piezo amplifier and drives the clamped piezo shaker, which generates the mechanical excitation of the cantilever in resonance. Due to the higher mass of the shaker, the phase of FG–CH1 is chosen as the reference and the phase of FG–CH2 is varied accordingly. The Wheatstone bridge is powered by FG–CH2 where the cantilever acts as device under test (DUT). In literature the piezoelectric sensor is modelled as a high-pass filtered open-circuit voltage, which will be amplified with a high impedance buffer for cantilevers [27] and membranes [28]. Beside this, the electrical impedance behavior of resonators with large Q-factors are described with the well-established extended Butterworth Van-Dyke equivalent circuit [29]. Its magnitude and phase is electrically read out by a differential amplifier (∆*U*) and recorded by the oscilloscope (OSC) at OSC–CH2. As verification of the electrical measurements, the oscillation amplitude of the cantilever is measured with a Micro System Analyzer (MSA-400, Waldbronn, Germany) from Polytec and converted to a velocity proportional voltage signal, which is recorded by the oscilloscope at OSC–CH1.

**Figure 5:**
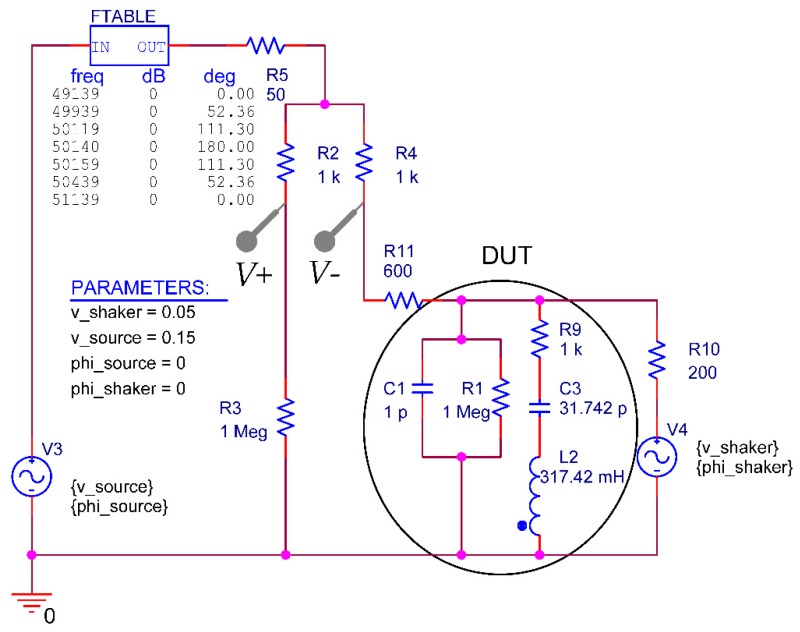
Equivalent circuit of the electrical circuit including the piezoelectric cantilever, the Wheatstone bridge and the shaker which includes the phase lag of the stimulating voltage at the integrated piezoelectric layer and the shaker impact.

A circuit simulation with PSpice of the electrical circuit is performed to verify the electrical measurements at the oscilloscope (see Figure 5), which includes the description of the electrical impact of the mechanical shaker on the differential voltage (∆*U = V_+_* − *V*_−_).

The simulation circuit is powered with two voltage sources (V3 and V4). V3 (internal resistor R5) powers the Wheatstone bridge (WB) which consists of R2, R3, R4 and the DUT. V4 represents the impact of the shaker on ∆*U.* The variation of the phase lag of V3 is reached by the block FTABLE which generates the frequency-dependent linear interpolated phase shift. The piezoelectric cantilever (DUT) is modelled with parasitic components R1, C1 and a series resonant circuit R9, C3, L2 and the component characteristics are calculated based on the theoretical formulas. Finally, R11 and R10 models the wiring between the WB and the DUT and the impact of the shaker that are determined experimentally by adjusting the voltage baseline and the resonance peak amplitude.

## 3. Results and Discussion

**Figure 6:**
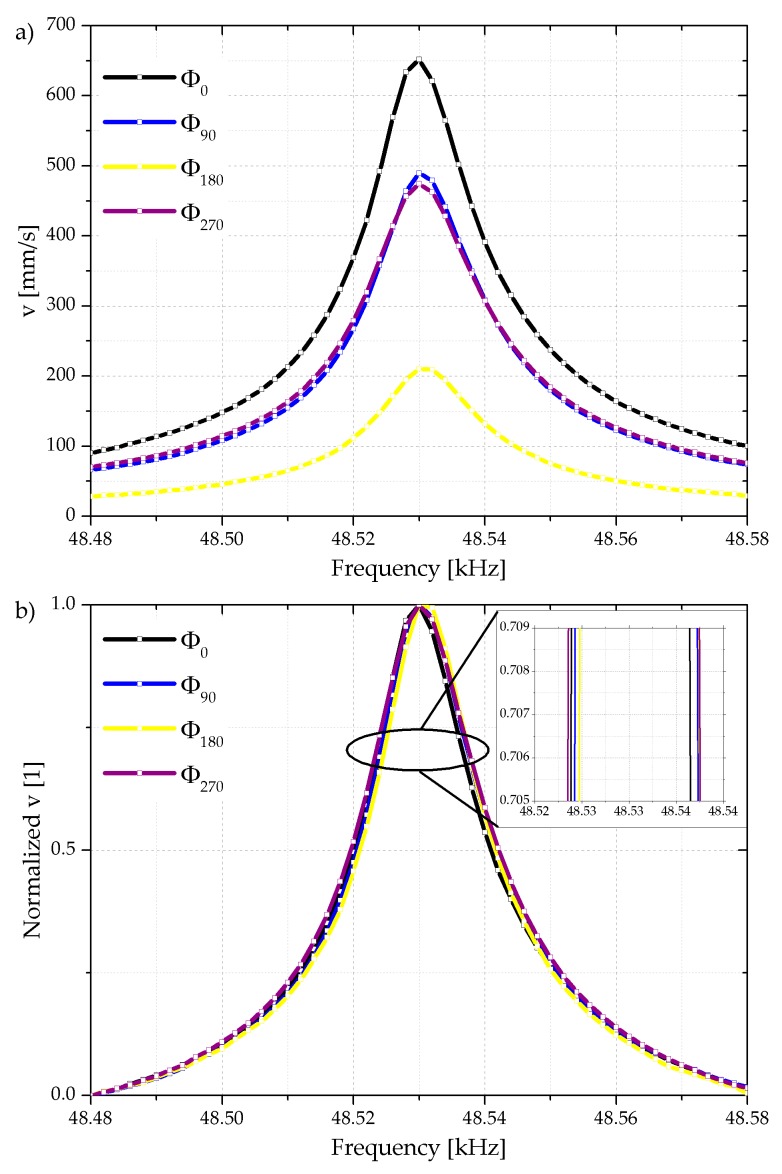
Selected examples of optically measured absolute velocity (**a**) and normalized velocity profiles measured at the cantilever tip (**b**) as a function of cantilever actuation phase shifts ranging from 0° to 270° with respect to the shaker actuation. The inserted lines represents a phase shift of 0°, 90°, 180°, and 270° and serve as guide to the eyes.

Before using the frequency dependent phase shift approach, the independence of the Q-factor with respect to any frequency independent constant phase shift between cantilever and shaker actuation is verified in air atmosphere. The power supply of the shaker at FG–CH1 acts as the reference phase while the phase of the integrated cantilever actuation is changed in 30° steps from 0° to 330°. The oscillation of the cantilever is recorded optically with a laser Doppler vibrometer (LDV) and electrically by the Wheatstone bridge and the differential voltage amplifier, respectively. Basically, the cantilever oscillation amplitude depends on the cantilever actuation phase and selected phases are shown in Figure 6a. When increasing the phase shift the amplitude of the cantilever oscillation is reduced reaching its lowest value at 180° due to destructive interference. Considering the Q-factor, the normalized cantilever velocity is presented in Figure 6b, indicating due to equal curve shapes an almost constant Q-factor in the range of 3460 to 3860 independent of the phase shift applied to the integrated piezoelectric thin film actuator.

**Figure 7:**
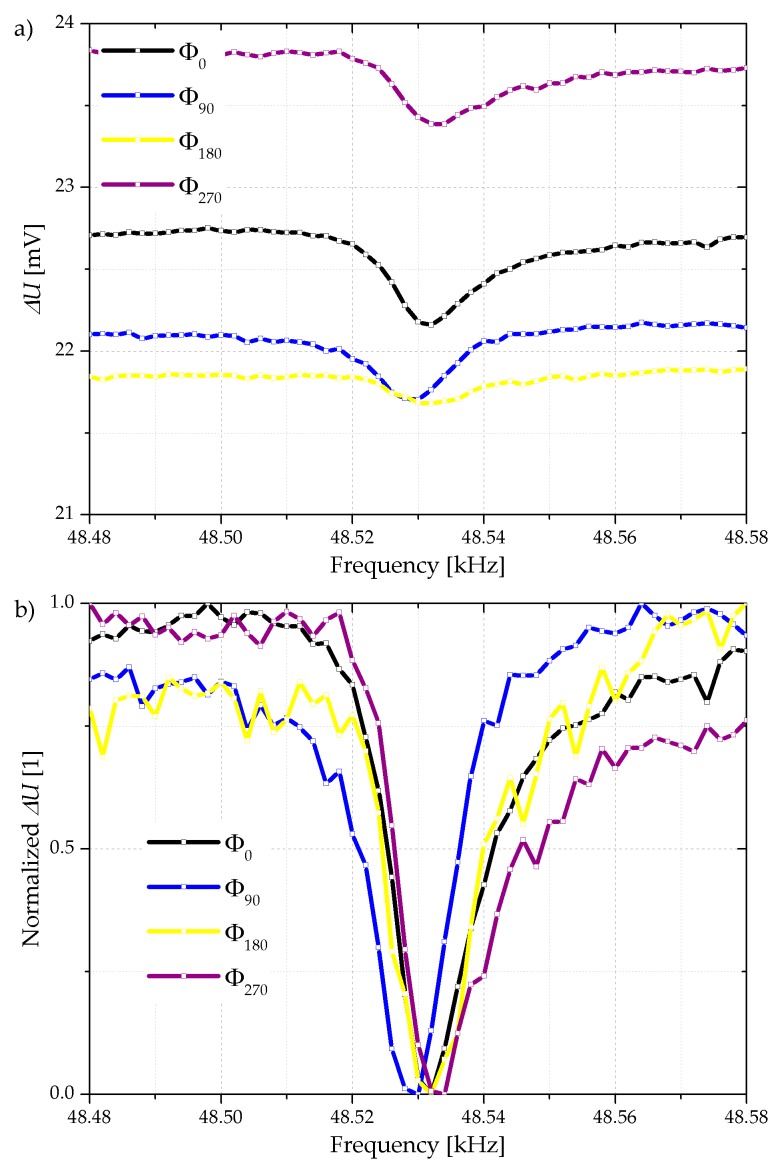
Selected examples of frequency-dependent Wheatstone bridge differential voltage profiles (**a**) and corresponding normalized values (**b**). The cantilever actuation phase shift varied stepwise between 0° and 270° with respect to the shaker actuation.

At the same time the electrical measurements of ∆*U* at the Wheatstone bridge show a cantilever resonance signal as illustrated in Figure 7a. Here the phase shift has the same effect on the amplitude of the cantilever oscillation, which results in a lower electrical amplitude of ∆*U* at resonance frequency. If these curves are normalized where minimal values are set to 0 and the resulting curves normalized to 1 as shown Figure 7b, a straightforward comparability of the electrically measured Q-factors which are ranging between 3300 and 5300 is possible.

**Figure 8:**
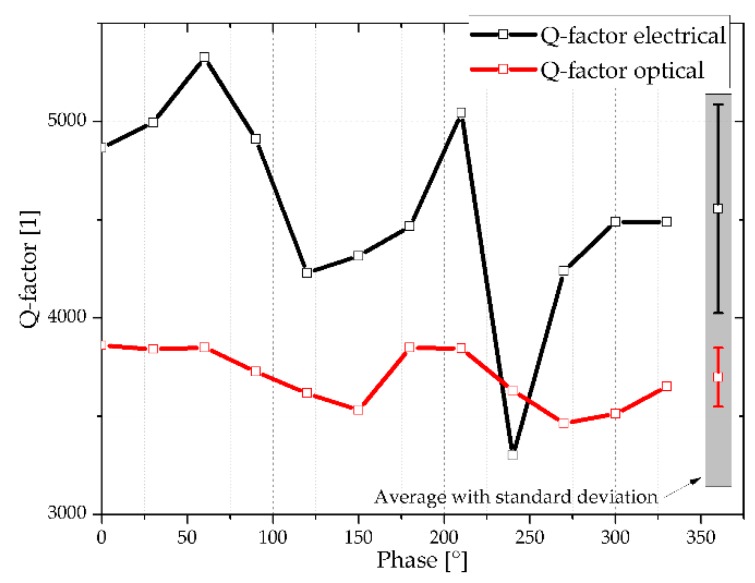
Q-factor characteristics determined either electrically or optically as a function of phase shift between cantilever actuation and shaker. The inserted lines serve as guide to the eyes.

The large noise of the electrical output signal is due to the non-shielded wiring circuit and due to the DC-related fraction of the electrical signal which prevents the oscilloscope to make use of the entire measurement range. The Q-factors are determined by $Q=\frac{f_{r}}{B_{0.707}}$ (optical measurement) or $Q=\frac{f_{r}}{B_{0.293}}$ (electrical measurement), where $f_{r}$, *B*_0.707_ and *B*_0.293_ represent the resonance frequency and amplitude bandwidths for both LDV and electrical measurements, respectively. As shown in Figure 8, the Q-factors determined by LDV are constant within the measurement accuracy of about ±6%, but those deduced from the electrical measurements show a substantially higher noise due to the poor peak characteristics in resonance. The constant Q-factor arises from the fact that the absolute oscillating amplitude depends on the phase between shaker and cantilever actuation, but the relative change of the oscillating amplitude remains the same, hence the constructive or destructive superimposition has no influence on the Q-factor.

**Figure 9:**
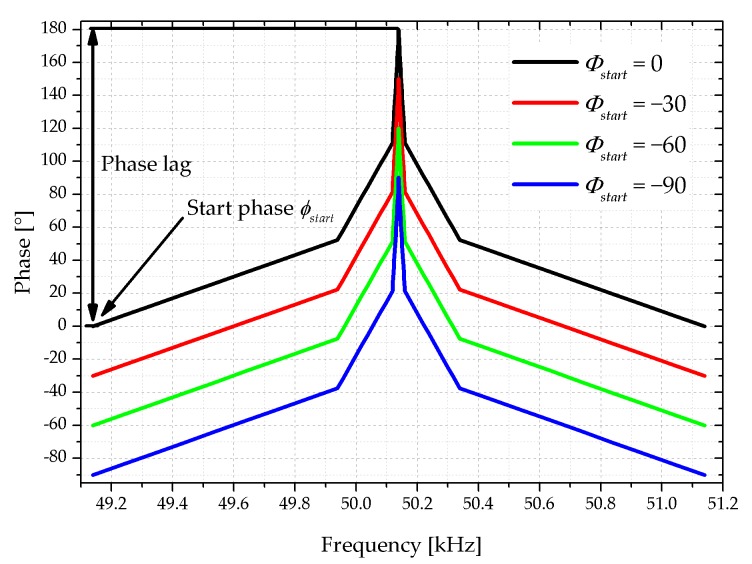
Phase characteristics of the stimulating voltage at the integrated piezoelectric layer with respect to the supply voltage of the shaker. The phase lag is kept constant while the starting phase *φ_start_* is varied to manipulate the Q-factor.

Based on the previous result, where any constant phase shift does not affect the Q-factor, an active Q-factor adjustment with two parameters is defined, namely the phase lag and the start phase *φ_start_*. Here the frequency dependent phase shift approach is introduced. The proposed phase profile is symmetric around the resonance frequency, while the phase at resonance is increased to the maximum phase lag of 180°. The phase is chosen such that it approximates a typical impedance spectrum of a piezoelectric MEMS cantilever resonating in the first bending mode (see Figure 9). The second parameter is varied from −90° to 0°, which shifts the whole frequency-dependent curve, but does not change its shape.

**Figure 10:**
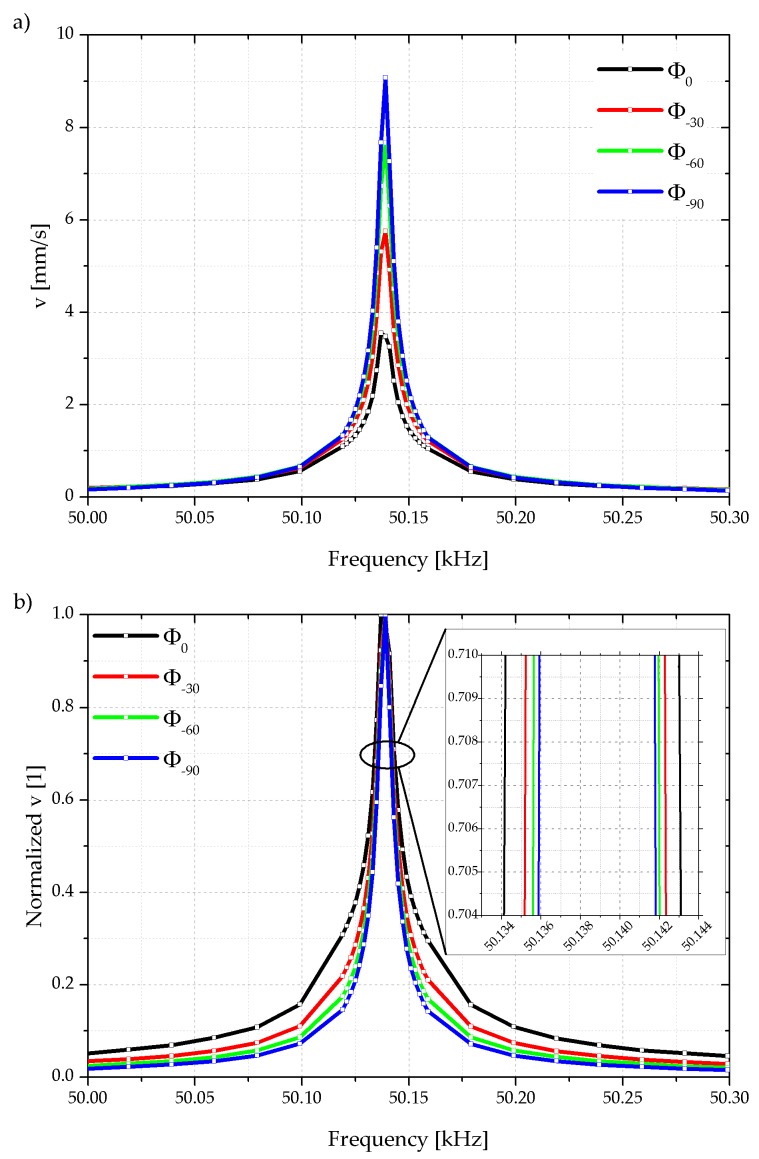
Optically measured absolute velocity (**a**) and normalized velocity of cantilever tip (**b**) when applying a frequency-dependent, phase shifted excitation. The inserted lines serve as guide to the eyes.

While the phase of the shaker actuation voltage at FG–CH1 retains at 0°, a MATLAB script constantly adjusts the phase shift at each individual frequency value of the integrated piezoelectric actuator, which is driven by FG–CH2. The oscilloscope OSC–CH1 records the cantilever tip oscillation measured with the laser Doppler vibrometer as shown in Figure 10a. The corresponding normalized oscillation amplitudes of the cantilever tip are shown in Figure 10b. Due to the change of *φ_start_* from −90° to 0° the cantilever oscillation amplitude responded with a broader normalized resonance peak which is shown in a more detailed view as insert in the figure.

**Figure 11:**
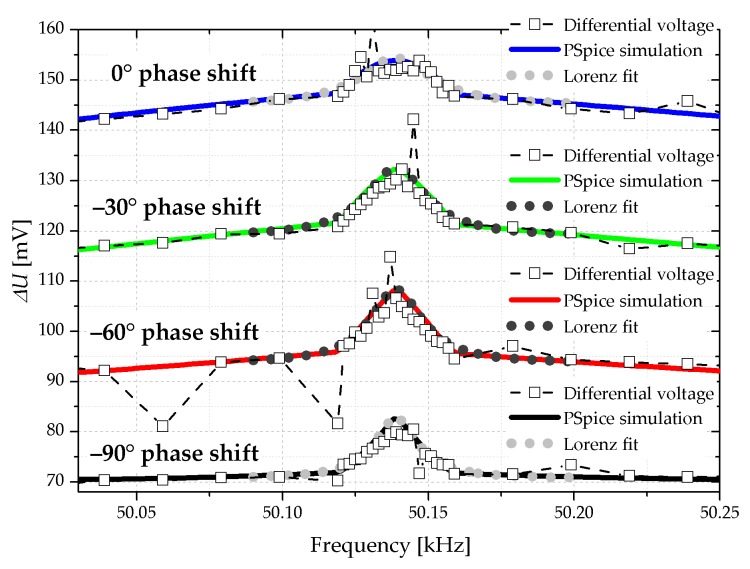
Comparison of the differential voltage of the Wheatstone bridge and the simulated voltage curve for the starting phase of −90° to 0°. For reasons of clarity, the individual curves are shifted of 20 mV.

Simultaneously the oscilloscope records the amplified electrical differential voltage at the Wheatstone bridge at OSC–CH2 and the frequency spectrum is shown in Figure 11. The figure shows the measured and the simulated values, whereas the latter are based on the equivalent circuit from Figure 5 and inserted with colored lines.

To demonstrate the impact of *φ_start_* on the Q-factor the values are extracted from Figure 10 and Figure 11 with a Lorenz fit and presented in Table 1.

**Figure 12:**
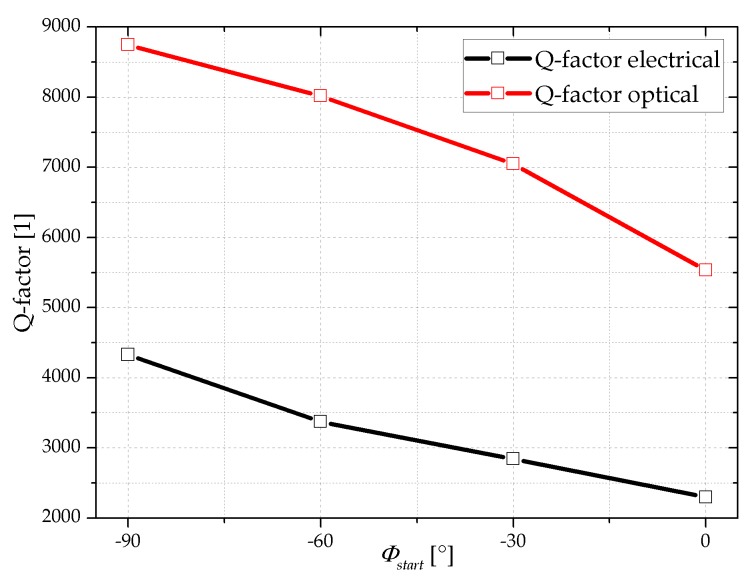
Q-factor characteristics determined either electrically or optically when applying a frequency-dependent, phase-shifted stimulus to the MEMS cantilever. The inserted lines serve as guide to the eyes.

The optically measured Q-factors change from 8746 at *φ_start_* = −90° to 5533 at *φ_start_* = 0° which is a reduction by a factor of about 1.6. The evaluation of the electrical spectrum reveals a Q-factor reduction from 4328 to 2299 which is a factor of about 1.9. The Q-factor values determined electrically or optically as a function of starting phase are shown in Figure 12.

Basically, the Q-factors determined electrically have less than half the value of those measured optically, independent of the stating phase value. This systematic deviation in Q-factors is attributed to the different measurement methods as the electrically measured Q-factor is influenced by the electrical circuit. In a small signal approximation the piezoelectric layer or the Wheatstone bridge may get short-circuited by the crosstalk through parasitic elements such as the piezoelectric layer and electrical wiring (see Figure 5).

The manipulation of the Q-factor arises from the adjustment of the phase lag between the shaker and cantilever in the observed frequency spectrum. That method enables a frequency dependent cantilever oscillation amplitude adjustment, where the amplitude far away from the resonance frequency is enlarged, whereas in resonance, it is decreased. Thereby the resonance peak is broadened, resulting in a lower Q-factor. Beneficial for a potential implementation is the variability of the phase characteristics of the stimulating voltage due to a large range of possible phase profiles.

## 4. Conclusions

In this study, an electronically tuneable reduction in Q-factor of MEMS cantilevers vibrating in the first bending mode is demonstrated by using an integrated piezoelectric thin film actuator. Based on a mechanical stimulation similar to those applied to excite standard AFM cantilevers, this approach for Q-factor tuning offers an easy-to-implement extension to existing AFM equipment. By electrically implementing the piezoelectric thin film actuator into a Wheatstone bridge configuration, the possibility is offered both for active manipulation and for measurement of the cantilever oscillation. When applying a frequency independent phase shift between the two actuators (i.e., shaker and on the cantilever), no impact on the Q-factor is observed within the measurement accuracy. As a consequence, a frequency dependent phase shift approach is introduced where the resonance behavior of a piezoelectric cantilever is mimicked. Based on the optical measurements a reduction of the Q-factor from 8746 down to 5533 is determined by changing the starting phase from −90° to 0°, which is a reduction by a factor of about 1.6. An electrical circuit simulation verifies the electrical readout of the Wheatstone bridge and demonstrates an electrical Q-factor manipulation of 4328 down to 2299, which represents a decrease by a factor of 1.9. The limited impact of this approach on the Q-factor compared to other damping techniques based on feedback controllers and automatically approximated phase shift is due to the straightforward detection principle. All in all, it is demonstrated that damping can be achieved in resonating MEMS devices with a tailored variation of the actuation phase requesting only standard, low-cost electronic equipment. In the near future, the integration of an on-chip compensation structure will reduce the parasitic effects of the resonance structure and enhance the Q-factor reduction when applying the phase shifted damping mode.

## Figures and Tables

**Table 1:** Table of Q-factors obtained by optical and electrical measurements when applying a frequency-dependent, phase-shifted stimulus to the MEMS cantilever.

*φ_start_*	Q_mech_	Q_el_
−90	8746	4328
−60	8016	3372
−30	7048	2843
0	5533	2299

## References

[1] Goeders K.M., Colton J.S., Bottomley L.A. (2008). Microcantilevers: Sensing chemical interactions via mechanical motion. Chem. Rev..

[2] Dionne E.R., Toader V., Badia A. (2014). Microcantilevers bend to the pressure of clustered redox centers. Langmuir.

[3] Tadigadapa S., Mateti K. (2009). Piezoelectric MEMS sensors: State-of-the-art and perspectives. Meas. Sci. Technol..

[4] Kucera M., Wistrela E., Pfusterschmied G., Ruiz-Diez V., Manzaneque T., Hernando-Garcia J., Sanchez-Rojas J.L., Jachimowicz A., Schalko J., Bittner A. (2014). Design-dependent performance of self-actuated and self-sensing piezoelectric-AlN cantilevers in liquid media oscillating in the fundamental in-plane bending mode. Sens. Actuators B Chem..

[5] Chu C.C., Dey S., Liu T.Y., Chen C.C., Li S.S. (2018). Thermal-Piezoresistive SOI-MEMS Oscillators Based on a Fully Differential Mechanically Coupled Resonator Array for Mass Sensing Applications. J. Microelectromech. Syst..

[6] Wasisto H.S., Merzsch S., Uhde E., Waag A., Peiner E. (2015). Handheld personal airborne nanoparticle detector based on microelectromechanical silicon resonant cantilever. Microelectron. Eng..

[7] Michels T., Rangelow I.W. (2014). Review of scanning probe micromachining and its applications within nanoscience. Microelectron. Eng..

[8] Boisen A., Dohn S., Keller S.S., Schmid S., Tenje M. (2011). Cantilever-like micromechanical sensors. Rep. Prog. Phys..

[9] Ababneh A., Schmid U., Hernando J., Sánchez-Rojas J.L., Seidel H. (2010). The influence of sputter deposition parameters on piezoelectric and mechanical properties of AlN thin films. Mater. Sci. Eng. B.

[10] Pérez-Campos A., Iriarte G.F., Hernando-Garcia J., Calle F. (2015). Post-CMOS compatible high-throughput fabrication of AlN-based piezoelectric microcantilevers. J. Micromech. Microeng..

[11] Ambacher O. (1998). Growth and applications of Group III-nitrides. J. Phys. D Appl. Phys..

[12] Pfusterschmied G., Kucera M., Steindl W., Manzaneque T., Ruiz Díez V., Bittner A., Schneider M., Sánchez-Rojas J.L., Schmid U. (2016). Roof tile-shaped modes in quasi free–free supported piezoelectric microplate resonators in high viscous fluids. Sens. Actuators B Chem..

[13] Kucera M., Hofbauer F., Wistrela E., Manzaneque T., Ruiz-Díez V., Sánchez-Rojas J.L., Bittner A., Schmid U. (2014). Lock-in amplifier powered analogue Q-control circuit for self-actuated self-sensing piezoelectric MEMS resonators. Microsyst. Technol..

[14] Hemon S., Akjouj A., Soltani A., Pennec Y., El Hassouani Y., Talbi A., Mortet V., Djafari-Rouhani B. (2014). Hypersonic band gap in an AlN-TiN bilayer phononic crystal slab. Appl. Phys. Lett..

[15] Maurício M.D.L., Paulo V.S. (2005). Modulation of photonic structures by surface acoustic waves. Rep. Prog. Phys..

[16] Mayrhofer P.M., Rehlendt C., Fischeneder M., Kucera M., Wistrela E., Bittner A., Schmid U. (2017). ScAlN MEMS Cantilevers for Vibrational Energy Harvesting Purposes. J. Microelectromech. Syst..

[17] Steven R.A., Henry A.S. (2007). A review of power harvesting using piezoelectric materials (2003–2006). Smart Mater. Struct..

[18] Fairbairn M.W., Moheimani S.O.R., Fleming A.J. (2011). Control of an Atomic Force Microscope Microcantilever: A Sensorless Approach. J. Microelectromech. Syst..

[19] Eaton P., West P. (2010). Atomic Force Microscopy.

[20] Chen L., Yu X., Wang D. (2007). Cantilever dynamics and quality factor control in AC mode AFM height measurements. Ultramicroscopy.

[21] Fairbairn M.W., Muller P., Moheimani S.O.R. (2014). Sensorless Implementation of a PPF Controller for Active Q Control of an AFM Microcantilever. IEEE Trans. Control Syst. Technol..

[22] Coskun M.B., Alemansour H., Fowler A.G., Maroufi M., Moheimani S.O.R. (2018). Q Control of an Active AFM CantileverWith Differential Sensing Configuration. IEEE Trans. Control Syst. Technol..

[23] Ruppert M.G., Yong Y.K. (2017). Note: Guaranteed collocated multimode control of an atomic force microscope cantilever using on-chip piezoelectric actuation and sensing. Rev. Sci. Instrum..

[24] Fischeneder M., Oposich M., Schneider M., Schmid U. (2017). Tuneable Q-Factor of MEMS Cantilevers with Integrated Piezoelectric Thin Films. Proceedings.

[25] Fischeneder M., Wistrela E., Bittner A., Schneider M., Schmid U. (2017). Tailored wafer holder for a reliable deposition of sputtered aluminium nitride thin films at low temperatures. Mater. Sci. Semicond. Proc..

[26] Ababneh A., Al-Omari A.N., Dagamseh A.M.K., Qiu H.C., Feili D., Ruiz-Díez V., Manzaneque T., Hernando J., Sánchez-Rojas J.L., Bittner A. (2014). Electrical characterization of micromachined AlN resonators at various back pressures. Microsyst. Technol..

[27] Fleming A.J. (2013). A review of nanometer resolution position sensors: Operation and performance. Sens. Actuators A Phys..

[28] Yaghootkar B., Azimi S., Bahreyni B. (2017). A High-Performance Piezoelectric Vibration Sensor. IEEE Sens. J..

[29] Pettine J., Patrascu M., Karabacak D.M., Vandecasteele M., Petrescu V., Brongersma S.H., Crego-Calama M., Van Hoof C. (2013). Volatile detection system using piezoelectric micromechanical resonators interfaced by an oscillator readout. Sens. Actuators A Phys..

